# A metabolic vulnerability of small-cell lung cancer

**DOI:** 10.18632/oncotarget.25964

**Published:** 2018-08-17

**Authors:** Miyuki Nomura, Mami Morita, Nobuhiro Tanuma

**Affiliations:** Division of Cancer Chemotherapy, Miyagi Cancer Center Research Institute, Natori, Japan

**Keywords:** PKM1, PKM2, PKM, SCLC, metabolism

Small-cell lung cancer (SCLC) accounts for 15-20% of lung cancers, and patient prognosis in this subset is poorer than in other types of lung cancer. Genomic studies in a large cohort of SCLC patients revealed simultaneous inactivation of the tumor suppressors *TP53* and *RB1* but few driver mutations have been therapeutically targeted [1]. Therefore, patients with SCLC have not benefited from recent advances in targeted therapy. We recently revisited glucose metabolism in cancer and identified the glycolytic enzyme PKM1 as a potential new target for SCLC.

**Figure 1:**
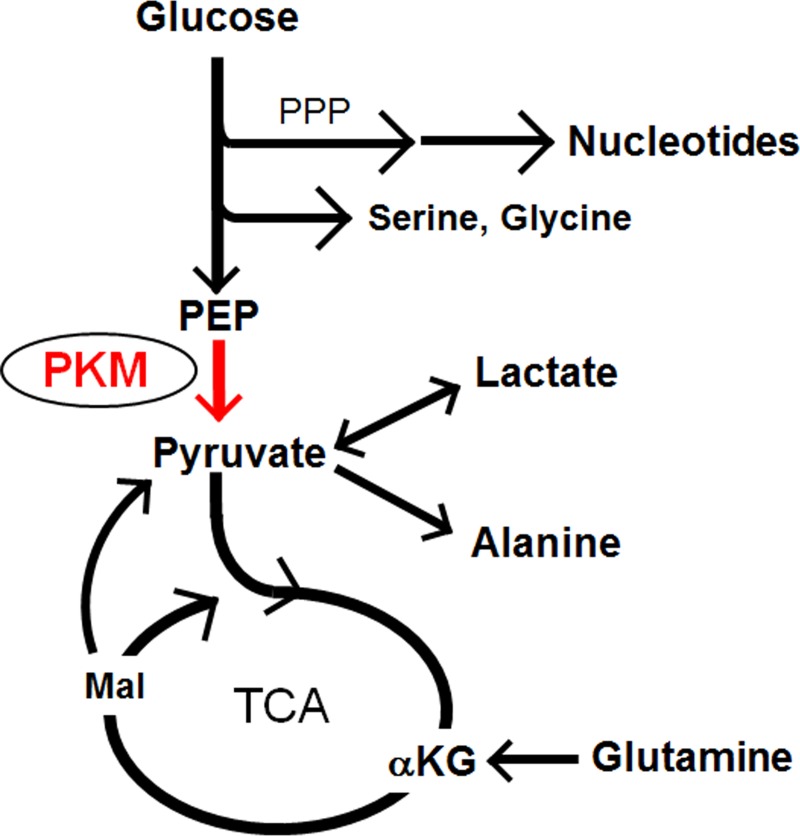
PKM at the crossroads of glucose, glutamine and lactate metabolism PPP, pentose-phosphate pathway; PEP, phosphoenol-pyruvate; TCA, tricarboxylic acid cycle; αKG, α-ketoglutarate; Mal, malate.

Enhanced glucose uptake is a cancer hallmark, as evidenced by the fact that most cancers including SCLC are effectively detected by a FDG-PET scan. Glucose taken up by tumor cells undergoes a series of glycolytic reactions to fuel other metabolic pathways such as the TCA cycle, the pentose-phosphate pathway and amino acid biosynthetic pathways (Figure 1). The end product of glycolysis is lactate, which was classically considered cellular waste. However, recent work reveals that lactate is not only released from but also actively taken up by cancer cells, particularly in *in vivo* settings, to fuel the TCA cycle after conversion back to pyruvate (see comment in reference [2]). It is also noteworthy that pyruvate can be generated from carbon source(s) other than glucose, such as glutamine. In these ways, glycolysis is linked to many metabolic pathways in an extensive and complex network.

The glycolytic pathway is comprised of 10 steps, three of which are irreversible. One is conversion of phosphoenol-pyruvate (PEP) to pyruvate, which is catalyzed by pyruvate kinase (PK). Vertebrates express 4 PK isozymes encoded by 2 genes, *PKM* and *PKLR*. *PKM* is predominant in most cells, whereas PKs in hepatocytes, red blood cells and some kidney cells are encoded by *PKLR*. Importantly, *PKM* transcripts exist as 2 splicing variants that give rise to distinct proteins: PKM1, which is constitutively active and promotes glucose catabolism, and PKM2, which is activated only in response to increased levels of allosteric activator(s) such as fructose 1,6-bisphosphate, an upstream intermediate in glycolysis. The latter property ensures that PKM2 will maintain a low rate of PEP-to-pyruvate conversion from glucose relative to PKM1. Generally, expression of PKM1 and PKM2 is mutually exclusive in a given cell type.

PKM isoform switching has received much attention in cancer metabolism since the discovery that PKM2, but not PKM1, is highly expressed in cancer cells and apparently favors tumor growth in some cell lines [3]. One conclusion was that high, non-regulatable PKM1 activity is not compatible with proliferation for unknown reasons, and thus, that PKM2 is the predominant isoform in dividing cells. Paradoxically, however, PKM2-knockout (KO) mice, which are deficient only in a PKM2-specific exon, show enhanced rather than decreased tumorigenesis [4]. Interestingly, PKM2-KO mice also display compensatory and partial expression of the more active isoform PKM1, although at varying levels [4]. In these contexts, it has been unclear whether PKM2 promotes or suppresses tumor growth.

To address these questions, we developed two lines of knock-in (KI) mice, each expressing either PKM1 or PKM2 [5]. Mice from both lines developed normally and were fertile, strongly suggesting that PKM1 expression itself did not significantly perturb normal cell proliferation or differentiation. More importantly, evaluation of our models revealed that relatively high PKM1 activity, as compared to PKM2, confers metabolic advantages and promotes tumor growth in various experimental models. PKM1-expressing cells exhibited both higher glucose flux into the TCA cycle and a higher rate of glucose conversion to lactate. Recent studies have revised ideas relevant to glucose metabolism and cancer: number of studies report that tumor cells show higher glycolytic and TCA cycle-related activities than do their normal counterparts (reviewed in reference [6]). These findings contrast with previously proposed models reporting a switch from oxidative to non-oxidative glucose metabolism in tumor cells. The PKM1-dependent metabolic changes (relative to PKM2) that we observe resembled the new view of metabolic reprograming reported in cancer. Some have suggested non-metabolic functions for PKM2 (reviewed in reference [7]) but those have not yet been confirmed in our models so far and may be context-dependent. We also report that PKM1 did not impede biosynthetic glucose metabolism through the pentose phosphate pathway. Finally, our results also provide an alternate mechanism for accelerated tumorigenesis seen in PKM2-KO mice, a phenotype previously explained by non-cell autonomous mechanisms involving dysregulation of systemic glucose homeostasis [4].

This work supports the idea that PKM1 boosts tumor cell growth cell-intrinsically by activating glucose metabolism. However, to date few tumors were known to express PKM1. Our new study reported the discovery that tumor cells in SCLC, the most malignant type of lung cancer, express PKM1 at high levels [5]. Cells of origin likely influence pathway preferences in tumor cells [8]. We have now reported that bronchial neuroendocrine cells, which give rise to SCLC, were PKM1- rather than PKM-2 positive [5, 9]. Moreover, we observed that PKM1, but not PKM2, can fully support survival and/or proliferation of human SCLC cell lines, highlighting PKM1 and factors related its activity as potential targets to treat SCLC. We are now developing small- or middle-sized molecules to target PKM1 directly or perturb PKM1-PKM2 splicing.

Our new findings also indicate that PKM1 activates autophagy in transformed MEFs [5], although how PKM1 favors SCLC tumor cell growth mechanistically remains to be addressed. SCLC cell dependency on high PK activity appears much greater than that of other cancer types, suggesting that SCLC cells have unique metabolic properties and are particularly vulnerable to down-modulation of PK activity. Relevant to this, Huang F. et al recently reported an interesting metabolic feature of SCLC, namely, inosine monophosphate dehydrogenase dependence, although this property was only seen in a minor subset (ASCL1^low^) of these cancers [10]. Better understanding of SCLC cell metabolism is needed to devise novel therapeutic strategies to treat this aggressive malignancy, which harbors few druggable mutations.
